# Determination of the α/β ratio for the normal liver on the basis of radiation-induced hepatic toxicities in patients with hepatocellular carcinoma

**DOI:** 10.1186/1748-717X-8-61

**Published:** 2013-03-15

**Authors:** Seok Hyun Son, Hong Seok Jang, Hyochun Lee, Byung Ock Choi, Young Nam Kang, Jeong Won Jang, Seung Kew Yoon, Chul Seung Kay

**Affiliations:** 1 Department of Radiation Oncology, Incheon St. Mary’s Hospital, College of Medicine, the Catholic University of Korea, Incheon, Korea; 2 Department of Radiation Oncology, Seoul St. Mary’s Hospital, College of Medicine, the Catholic University of Korea, Seoul, Korea; 3 Department of Internal Medicine, Incheon St. Mary’s Hospital, College of Medicine, the Catholic University of Korea, Incheon, Korea; 4 Department of Internal Medicine, Seoul St. Mary’s Hospital, College of Medicine, the Catholic University of Korea, Seoul, Korea

**Keywords:** Radiation-induced hepatic toxicity, An increase in Child-Pugh score, Helical tomotherapy

## Abstract

**Background:**

The purpose of this study was to determine the α/β ratio for normal liver with hepatitis by analyzing the toxicity data from patients with unresectable hepatocellular carcinoma treated with helical tomotherapy.

**Methods:**

Between March 2006 and February 2012, 98 patients were eligible for this study. 66 patients received 45–50 Gy in 4.5-5 Gy fractions (Group A) and 32 patients received 36–60 Gy in 2.5-3 Gy fractions (Group B). Radiation-induced hepatic toxicity was defined as an increase of at least 2 points in the Child-Pugh score within 4 months of completing helical tomotherapy. We attempted to find the statistically significant parameters in the 2 groups using α/β ratios of 2, 4, 6, 8, or 10, and compared the estimated probability curves of each significant parameter. We hypothesized that the α/β ratio associated with the best matches for the curves between the 2 groups would be equivalent to the α/β ratio for the normal liver.

**Results:**

When using an α/β ratio of 2 or 4, different parameters were found to be statistically significant in a multivariate analysis (Group A: V_BED30_ for α/β ratio = 2 and V_BED25_ for α/β ratio = 4, Group B: V_BED25_ for α/β ratio = 2 and V_BED20_ for α/β ratio = 4). When using an α/β ratio of 6, 8, or 10, V_BED20_ was found to be a statistically significant parameter in both groups. Comparison of the estimated probability curve of each significant parameter between the groups revealed that an α/β ratio of 8 resulted in the best matches.

**Conclusions:**

We suggest that the α/β ratio of the normal liver with hepatitis is 8. We hope that previously reported parameters and their values can be effectively used in different fractionation schemes by calculating the biologically effective dose using an α/β ratio of 8.

## Background

Radiation-induced hepatic toxicity (RIHT) is a serious dose-limiting toxicity in patients with hepatocellular carcinoma (HCC) receiving radiotherapy (RT) because there is currently no effective treatment for RIHT, which can ultimately cause liver failure [1]. Several studies have shown that there are a number of predictive parameters for RIHT, and these have helped to reduce hepatic toxicity associated with the use of RT [2-11].

When treating patients with different fraction size compared to those of previous studies, the expected tumor response and hepatic toxicity could be estimated by calculating the biologically effective dose (BED) according to the linear-quadratic model. An α/β ratio of 10 is commonly used to calculate the BED delivered to the tumor, but the BED for the normal liver could not be calculated as its α/β ratio was unknown. In previous studies on hepatic toxicity, an α/β ratio of 2–3 or 10 was used to analyze the toxicity data arising from different fraction sizes and total doses of radiation [2,9,10].

In addition, the majority of patients with HCC have hepatitis, unlike patients with metastatic liver tumors, especially in eastern countries. In patients with HCC, the tolerance of the normal liver to radiation was considerably reduced [12]. Determining the α/β ratio of the normal liver with hepatitis is important to improve the tumor control and prevent the hepatic toxicity resulting from the use of RT in HCC patients.

In this study, we attempt to determine the α/β ratio for the normal liver with hepatitis by analyzing the hepatic toxicity data from patients treated with 4.5-5 Gy per fraction (Group A) and 2.5-3 Gy per fraction (Group B), respectively, and comparing the 2 groups.

## Methods

### Patients

The inclusion criteria for this study were as follows: 1) unresectable locally advanced HCC, 2) prior treatment with hypofractionated helical tomotherapy with a curative aim, 3) a follow-up period of 4 months, 4) at least 2 laboratory studies within 4 months of completing helical tomotherapy, 5) at least 2 radiologic study within 4 months of completing helical tomotherapy, and 6) no intrahepatic disease progression within 4 months of completing helical tomotherapy.

Between March 2006 and February 2012, a total of 98 patients were found to meet these inclusion criteria. All of the patients received hypofractionated RT using TomoTherapy Hi-Art (TomoTherapy, Madison, WI, USA) at Seoul St. Mary’s Hospital and Incheon St. Mary’s Hospital, the Catholic University of Korea. Of those, 66 patients received 45–50 Gy in 4.5-5 Gy fractions (Group A) and 32 patients received 36–60 Gy in 2.5-3 Gy fractions (Group B). The patients’ clinical and dosimetric data were retrospectively collected following Institutional Review Board approval (IRB of Incheon St. Mary's Hospital, the Catholic University of Korea, Reference number: OC12RISI0062). The patients’ characteristics are shown in Table 1.

**Table 1:** Clinical characteristics of patients in Group A and Group B

**Characteristics**	**Group A (n = 66)**		**Group B (n = 32)**		***p *value**
	**n**	**%**	**n**	**%**	
Gender					0.147
Male	52	78.8	29	90.6	
Female	14	21.2	3	9.4	
Age (year)					0.433
Median	61		56		
Range	40-80		40-78		
ECOG PS					0.351
0	22	33.3	10	31.3	
1	44	66.7	21	65.6	
2	0	0	1	3.1	
Hepatitis					0.484
HBV	47	71.2	24	75.0	
HCV	6	9.1	2	6.3	
Others	13	19.7	6	18.9	
Liver cirrhosis					0.279
No	14	21.2	10	31.2	
Yes	52	78.8	22	68.8	
PVTT					0.677
No	28	42.4	15	46.9	
Yes	38	57.6	17	53.1	
AFP (IU/mL)					0.190
<400	45	68.2	18	56.3	
≥400	21	31.8	14	43.7	
Child-Pugh class					0.059
A	49	74.2	29	90.6	
B	17	25.8	3	9.4	
AJCC stage					0.741
II	12	18.2	4	12.5	
III	47	71.2	25	78.1	
IV	7	10.6	3	9.4	
Previous treatment					0.964
No	7	10.6	3	9.4	
Yes	59	89.4	29	90.6	
TACE	58	87.9	28	87.5	
RFA	7	10.6	2	6.3	
PEI	7	10.6	3	9.4	
Surgery	9	13.6	7	21.9	
Treatment after RT					0.180
No	31	47.0	10	31.3	
Yes	35	53.0	22	68.8	
TACE	35	53.0	22	68.8	
RFA	2	3.0	0	0	
PEI	2	3.0	1	3.1	
Systemic CTx	3	4.5	3	9.4	

Abbreviations: ECOG PS = Eastern Cooperative Oncology Group performance status; HBV = hepatitis B virus; HCV = hepatitis C virus; PVTT = portal vein tumor thrombosis; AFP = alpha-fetoprotein; AJCC = American Joint Committee on Cancer; TACE = transcatheter arterial chemoembolization; RFA = radiofrequency ablation; PEI = percutaneous ethanol injection; RT = radiotherapy; CTx = chemotherapy.

### Target volume and treatment

The gross tumor volume (GTV) was defined as that which was enhanced in the arterial phase and diluted in the delayed phase of the computed tomography (CT) scan. The planning target volume (PTV) was generated by adding 5–15 mm to the GTV in 63 of the 98 patients, facilitating asymmetric margin expansion to reduce irradiation to the stomach, duodenum, and small intestine. In the remaining 35 of the 98 patients, 4-dimensional CT (4D-CT) was performed to generate the internal target volume to compensate for respiration-induced liver movement because of the installation of 4D-CT in March 2009 at Seoul St. Mary’s hospital and in March 2011 at Incheon St. Mary’s hospital. Organs at risks such as the total liver, non-target normal liver (NTNL), stomach, duodenum, intestine, kidney, and spinal cord were also contoured for evaluation of the irradiated dose. The NTNL volume was the total liver volume minus the PTV.

The radiation dose was 45–50 Gy in 4.5-5 Gy fractions for Group A and 36–60 Gy in 2.5-3 Gy fractions for Group B, prescribed to 95% of the PTV. 4.5-5 Gy per fraction was used in smaller PTV and 2.5-3 Gy per fraction was used in larger PTV. Prior to actual beam delivery, megavoltage cone-beam CT was performed during every treatment session. The patients’ set-up and position were corrected using automated image registration, and the anatomical accuracy was always evaluated by a radiation oncologist.

### Definition and evaluation of the radiation-induced hepatic toxicity

RIHT was defined as an increase of at least 2 points in the Child-Pugh score (CP score) within 4 months after the completion of helical tomotherapy. The CP score, which is calculated on the basis of the serum bilirubin and albumin levels, the prothrombin time (PT), and the presence and degree of ascites or encephalopathy, is used as an assessment of hepatic function and an increase in the CP score reflects a deterioration in hepatic function [13].

Patients were evaluated weekly by a physician during the treatment and followed up every 1–2 months after the completion of treatment. At every visit, physical examinations and blood tests were performed to assess hepatic toxicity. The levels of aspartate transaminase (AST), alanine transaminase (ALT), alkaline phosphatase (ALP), serum albumin, total bilirubin and the PT were examined and we also checked the presence of ascites and hepatic encephalopathy.

### Determination of the α/β ratio for the normal liver

**Figure 1:**
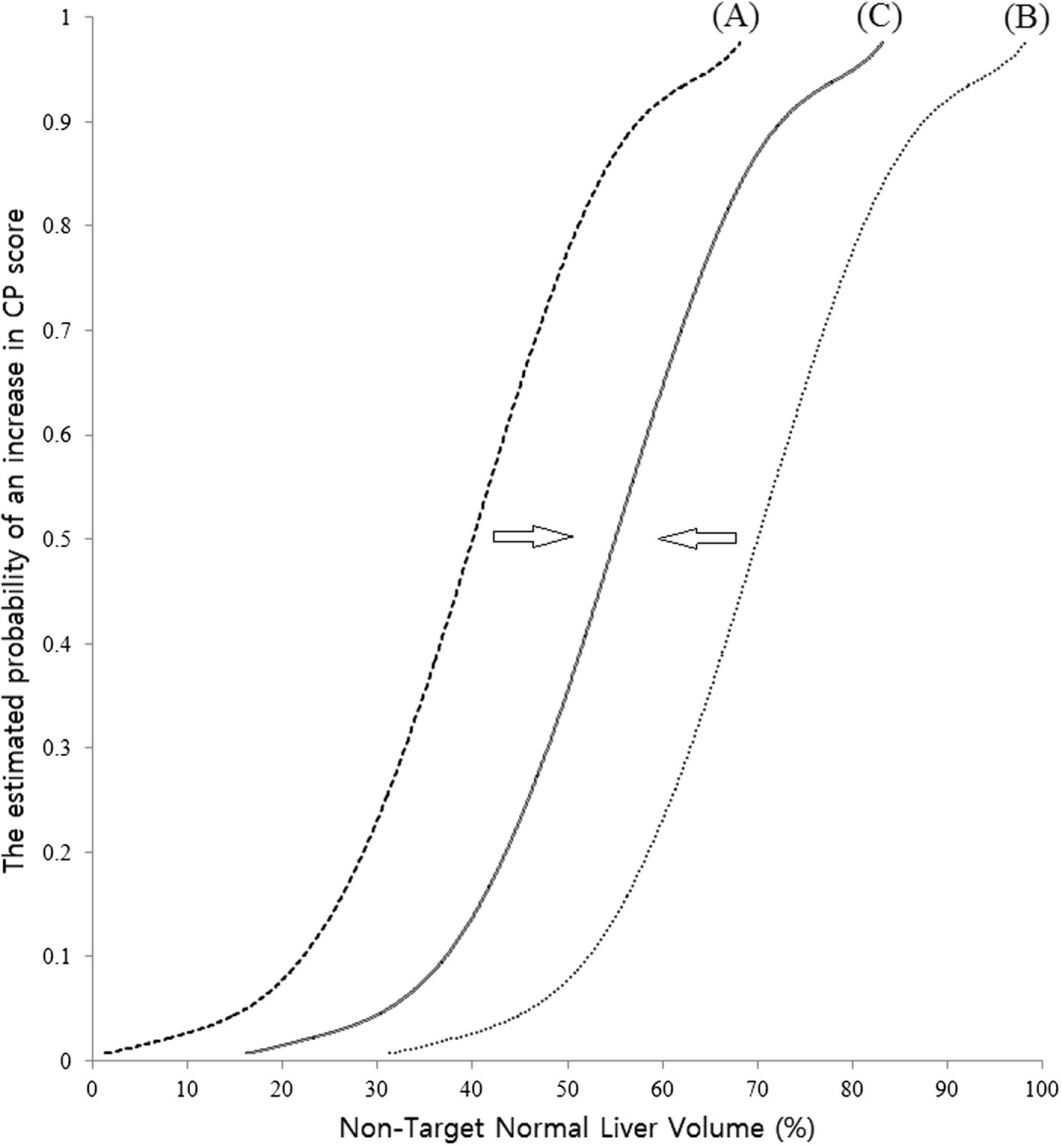
**A schematic explanation of our hypothesis.** (**A**, **B**) The estimated probability curves of statistically significant parameters in both groups using different fraction sizes. (**C**) The α/β ratio resulting in the best match for 2 curves between the groups will be equivalent to the α/β ratio for the normal liver.

We attempted to identify the statistically significant parameters in the 2 groups using an α/β ratio of 2, 4, 6, 8, or 10, and compared the estimated probability curves of each significant parameter (Figure 1A, 1B). We hypothesized that if the α/β ratio resulting in the best match for curves of each parameter between 2 groups would be equivalent to the α/β ratio of the normal liver (Figure 1C).

The dose-volumetric values were calculated on the basis of dose-volumetric histograms (DVH) and converted to the BED according to the linear quadratic model (assuming an α/β ratio of 2, 4, 6, 8, or 10, respectively) in all patients. The dose-volumetric parameters for predicting RIHT were the percentage of the NTNL volume receiving more than a BED of 5 Gy (V_BED5_), more than a BED of 10 Gy (V_BED10_), more than a BED of 15 Gy (V_BED15_), more than a BED of 20 Gy (V_BED20_), more than a BED of 25 Gy (V_BED25_), more than a BED of 30 Gy (V_BED30_), more than a BED of 35 Gy (V_BED35_), more than a BED of 40 Gy (V_BED40_), more than a BED of 45 Gy (V_BED45_) and more than a BED of 50 Gy (V_BED50_).

Using logistic regression models and receiver operating characteristic (ROC) curves, statistically significant parameters were identified in each group using an α/β ratio of 2, 4, 6, 8, or 10. The values of the area under the curve (AUC) were obtained for each α/β ratio group and each treatment group (Group A and Group B). We then compared the estimated probability curve of each significant parameter between Group A and Group B to determine the α/β ratio that resulted in the best match between the 2 curves.

### Statistical analysis

Pearson’s chi-square test and the independent t-test were used to compare the clinical characteristics between the 2 groups (Group A vs. Group B). Binary logistic analysis was used for univariate analysis of dose-volumetric parameters associated with RIHT. Multivariate analysis involved stepwise procedures containing all significant variables according to the univariate analysis, and the ROC curve was used to evaluate the significant dosimetric parameters. Statistical analysis was performed using SPSS ver. 12.0 (SPSS Institute, Chicago, Illinois) and a *p* value of <0.05 was considered to be significant.

## Results

A total of 98 patients were enrolled in this study. 66 patients received a daily fraction size of 4.5-5 Gy (Group A) and 32 patients received a daily fraction size of 2.5-3 Gy (Group B). There was no statistically significant difference between the 2 groups in terms of gender, age, Eastern Cooperative Oncology Group performance status (ECOG PS), pretreatment CP class, American Joint Committee on Cancer (AJCC) stage, the level of alpha-fetoprotein (AFP), the presence or absence of hepatitis, liver cirrhosis and portal vein tumor thrombosis (PVTT), prior treatment and treatment after RT (Table 1). The PTV for Group A and Group B were 134 ± 129 cm^3^ (mean ± standard deviation) and 382 ± 396 cm^3^, respectively, and the volume of the NTNL for Group A and Group B was 1301 ± 548 cm^3^ and 1184 ± 302 cm^3^, respectively.

**Figure 2:**
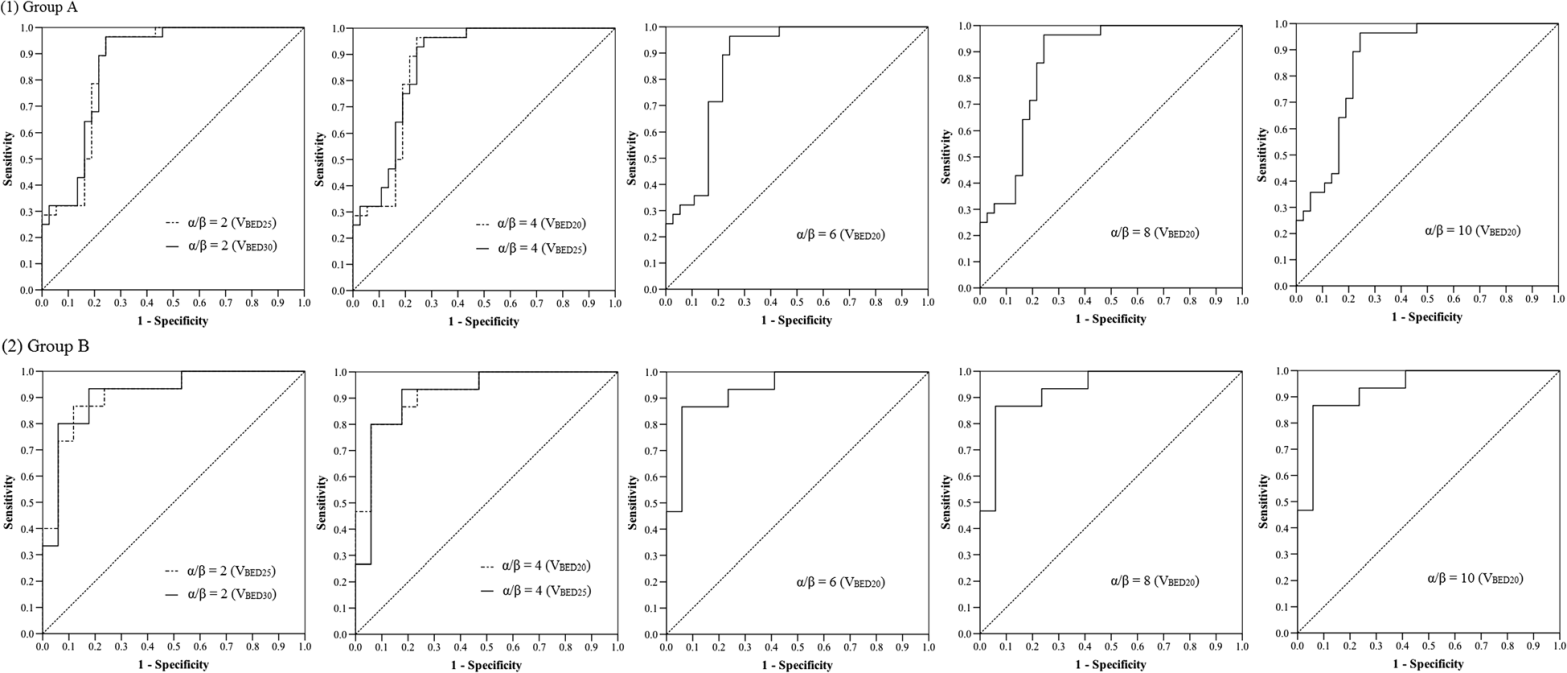
The ROC curves and derived AUC values for statistically significant parameters associated with an increase in the CP score in both groups using an α/β ratio of 2, 4, 6, 8, or 10.

After the completion of helical tomotherapy, RIHT developed in 43 (43.9%) of 98 patients: 28 patients (42.4%) in Group A and 15 patients (46.9%) in Group B. The results of multivariate analysis evaluating the association between dose-volumetric parameters and RIHT in each treatment group using an α/β ratio of 2, 4, 6, 8, or 10, are summarized in Table 2. When an α/β ratio of 2 or 4 was used, different parameters were statistically significant in multivariate analysis (Group A: V_BED30_ for α/β ratio = 2 and V_BED25_ for α/β ratio = 4, Group B: V_BED25_ for α/β ratio = 2 and V_BED20_ for α/β ratio = 4). Therefore, we compared the same parameters in each group using an α/β ratio of 2 or 4 (V_BED25_ and V_BED30_ for α/β ratio = 2 and V_BED20_ and V_BED25_ for α/β ratio = 4). When an α/β ratio of 6, 8, or 10 was used, V_BED20_ was a statistically significant parameter in both groups. In addition, these parameters were verified using the ROC curve and the values of AUC. These results are shown in Figure 2 and Table 2. The AUC values in group A and Group B were more than 0.861 and 0.914, respectively, which were statistically significant (*p* < 0.001).

**Table 2:** The significant parameters and the values of AUC associated with an increase in CP score in both groups according to α/β ratio of 2, 4, 6, 8, or 10

	**Group A**				**Group B**			
	**Significant parameter**	***p *value**	**AUC**	***p *value**	**Significant parameter**	***p *value**	**AUC**	***p *value**
†α/β = 2	V_BED25_	<0.001	0.859	<0.001	*V_BED25_	0.002	0.914	<0.001
	*V_BED30_	<0.001	0.863	<0.001	V_BED30_	0.003	0.914	<0.001
†α/β = 4	V_BED20_	<0.001	0.859	<0.001	*V_BED20_	0.002	0.922	<0.001
	*V_BED25_	<0.001	0.864	<0.001	V_BED25_	0.003	0.914	<0.001
α/β = 6	V_BED20_	<0.001	0.864	<0.001	V_BED20_	0.002	0.933	<0.001
α/β = 8	V_BED20_	<0.001	0.861	<0.001	V_BED20_	0.002	0.933	<0.001
α/β = 10	V_BED20_	<0.001	0.866	<0.001	V_BED20_	0.002	0.933	<0.001

*most significant parameter.

† When an α/β ratio of 2 or 4 was used, different parameters were statistically significant in multivariate analysis (Group A: V_BED30_ for α/β ratio = 2 and V_BED25_ for α/β ratio = 4, Group B: V_BED25_ for α/β ratio = 2 and V_BED20_ for α/β ratio = 4). Therefore, we compared same parameters in each group using an α/β ratio of 2 or 4 (V_BED25_ and V_BED30_ for α/β ratio = 2 and V_BED20_ and V_BED25_ for α/β ratio = 4).

Abbreviation: AUC = area under the curve.

**Figure 3:**
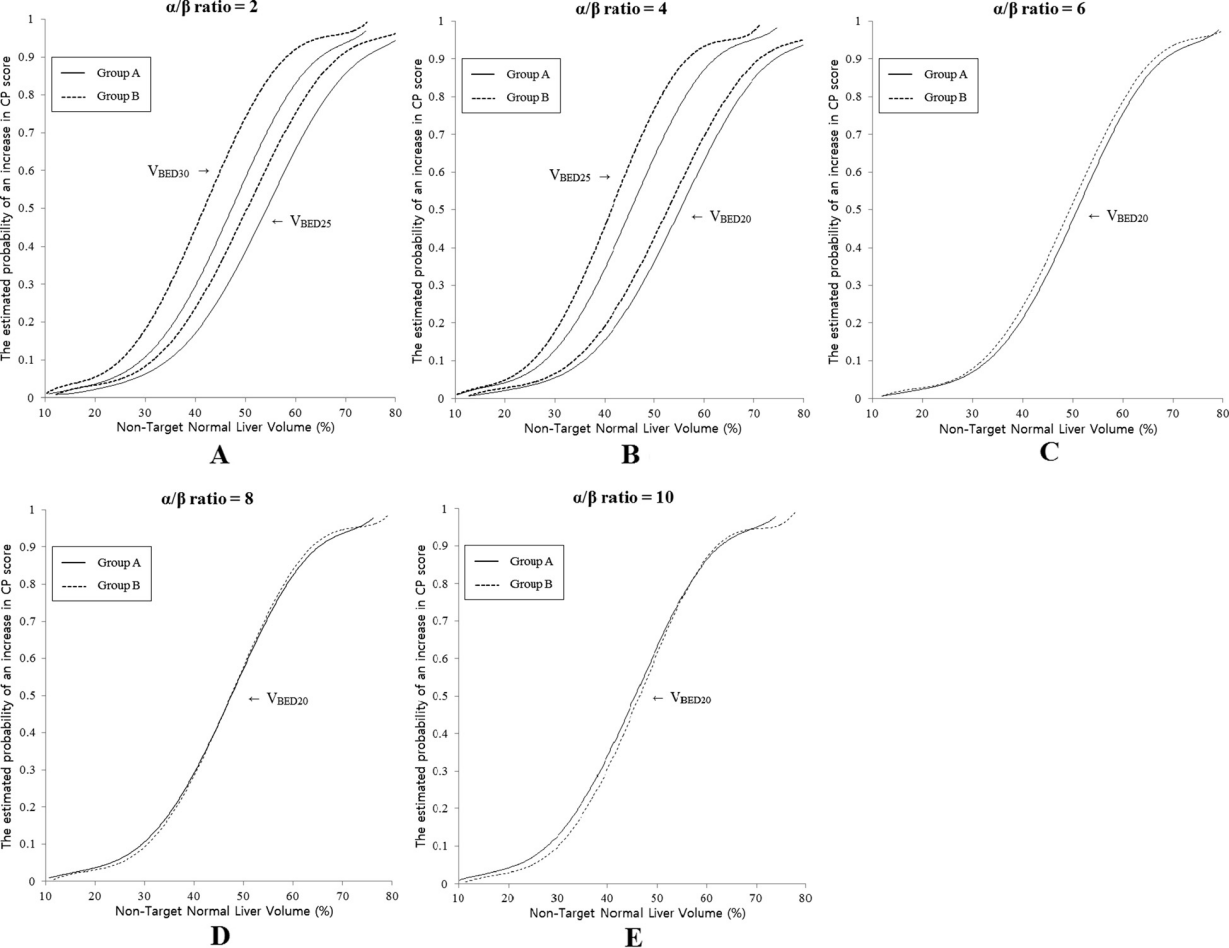
Comparison of the estimated probability curve of each significant parameter between groups to determine the α/β ratio for the normal liver.

Next, we compared the estimated probability curve of each significant parameter between the 2 groups to determine the α/β ratio for normal liver. According to the data in Figure 3, the estimated probability curves of significant parameters showed the closest match between the 2 groups using an α/β ratio of 8. Therefore, we suggest that the α/β ratio for the normal liver with hepatitis is 8.

## Discussion

RT has not been generally used for the treatment of HCC because of the low tolerable dose of radiation for whole liver, which is insufficient to achieve tumor control [14-16]. However, several studies have recently reported that partial volume irradiation of the liver is feasible and is a useful tool for the treatment of HCC within acceptable toxicity ranges [17-19]. Furthermore, the emergence of 3-dimensional treatment planning systems has enabled the collection of quantitative information regarding the dose-volume relationship in regions of interest, leading several investigators to report predictive parameters for RIHT [2-11]. These parameters and their values have helped to determine the appropriate radiation dose to be use in RT.

RIHT is a significant dose-limiting toxicity for RT in patients with HCC. Radiation-induced liver disease (RILD) is a well-established concept of hepatic toxicity [16], and previously, classic RILD was a serious hepatic toxicity caused by irradiation of 30–35 Gy to the entire liver. However, the incidence of classic RILD has decreased after partial volume irradiation came into general use [5,6]. Other authors reported the parameters predicting the non-classic RILD; for example, the elevation of hepatic enzymes ≥ grade 2 or 3 as defined by Radiation Therapy Oncology Group (RTOG) toxicity criteria or Common Terminology Criteria for Adverse Events (CTCAE) or the progression of CP class [2,5-8]. Although RIHT has been evaluated according to various different criteria, dose-volumetric parameters and their cut-off values resulting from these studies have been used as guidelines for radiation planning in the treatment of HCC.

There is some uncertainty when using different fraction sizes for treatment. Although an α/β ratio of 10 could be used to calculate the BED delivered to the tumor, the BED delivered to the normal liver could not be calculated as the α/β ratio for the normal liver was unknown. In spite of this uncertainty, various ranges of α/β ratio were used with respect to various different criteria of hepatic toxicity in previous studies. To calculate the BED delivered to the normal liver, Cheng *et al.* used an α/β ratio of 2 for grade 3 or worse CTCAE hepatic toxicity [10], and Dawson *et al.* used an α/β ratio of 2 or 2.5 for cases of classic RILD [3,9]. Kim *et al.* used an α/β ratio of 10 to calculate the BED for cases of grade 2 or worse CTCAE hepatic toxicity [2], and Liang *et al.* did not calculate the BED for classic RILD as there was no clear α/β ratio for the normal liver [4,5].

For these reasons, it is important to determine the α/β ratio for the normal liver. Here, we have attempted to determine the α/β ratio for the normal liver indirectly on the basis of the toxicity data from 2 patient groups treated with different fraction sizes.

We defined RIHT as an increase of at least 2 points in the CP score. In our previous study, the progression of CP class was analyzed as a useful radiation dose-limiting toxicity, whereas the elevation of hepatic enzymes according to the CTCAE scale was not appropriate in these circumstance [6]. Liaw *et al.* also used an increase of at least 2 points in the CP score to evaluate the deterioration of hepatic function in patients who were treated with lamivudine [13]. The CP score is an assessment tool to examine the severity of hepatic functional impairment, and the increase in the CP score reflects the deterioration in the hepatic function. Therefore, we believe that an increase in the CP score corresponds to the dose-limiting hepatic toxicity for the patients with HCC.

We analyzed the hepatic toxicities that occurred within 4 months of treatment. Classic RILD typically occurs 4–8 weeks after the completion of RT, although it has been reported to occur as early as 2 weeks and as late as 7 months [20]. According to Dawson *et al.* and Liang *et al.*, it occurred within 4 months after the completion of RT [3,5]. Kim *et al.* and Cheng *et al.* also reported that non-classic RILD hepatic toxicities occurred within 3 or 4 months [2,10]. Therefore, we considered a 4 month follow-up duration to be reasonable in this study.

In this study, 4.5-5 Gy per fraction was used in Group A and 2.5-3 Gy per fraction was used in Group B; The difference in fraction size between the 2 groups was thus 2 Gy. Although the fact that the same fraction size was not used for treatment was considered a weak point, the maximal difference in groups was only 0.5 Gy and it did not affect our analysis because the dose-volumetric values were converted to the BED according to the linear quadratic model (assuming α/β ratio of 2, 4, 6, 8, or 10) in all patients.

The results of our study suggest that the α/β ratio for the normal liver in the patients with HCC was 8, but there are a few points to be considered when accepting our result. First, the α/β ratio is derived from toxicity data when defining the RIHT as an increase of at least 2 points in the CP score. Whether our result could be applied equally when estimating classic RILD or non-classic RILD, such as RTOG or CTCAE hepatic toxicity grade, will need to be tested in further study. Because CTCAE grade was not an appropriate measure of dose-limiting hepatic toxicity, and classic RILD was not significant because of its rare incidence in our previous study, these types of toxicities were therefore not analyzed in this study [6]. Second, all the patients received helical tomotherapy. Because radiation is delivered continuously from all angles around the patient via a ring gantry, a much wider region of normal liver was irradiated with a low dose of radiation when compared to 3-dimensional conformal therapy. Our analysis showed the most significant parameters were V_BED20,_ V_BED25_ and V_BED30_, which were a lower level of dose-volumetric parameters than those of previous studies. This result reflects that it is due to the characteristic of the planning and delivery method of helical tomotherapy. Finally, all patients in this study had hepatitis (Table 1). The majority of patients with HCC also have hepatitis in eastern countries, and thus the liver is generally less tolerant of radiation [12]. According to Dawson *et al.*, the mean liver dose associated with a risk of RILD was higher for liver metastases than for primary HCC [3]. Therefore, when treating the metastatic liver tumors, an α/β ratio of 8 should be used with caution if used to calculate the BED for estimating RIHT.

Our study had some limitations. First, it was a retrospective study. Although there were no statistically significant differences between the 2 groups, confounding factors such as a selection bias might exist. Second, small number of patients were eligible for this study (66 patients in Group A and 32 patients in Group B). However, the analysis results were still reliable because the AUC values of statistically significant parameters were more than 0.859 and 0.914, respectively.

## Conclusion

We suggest that the α/β ratio for the normal liver with hepatitis is 8. We hope that previous reported parameters and their values can be effectively used in different fractionation schemes by calculating the BED using this α/β ratio, and we hope for further confirmation in larger-scale studies.

## Competing interests

The authors declare that they have no competing interests.

## Authors’ contribution

SHS, HL, HSJ, BOC and CSK performed all CT evaluations, target contouring, data collection and interpretation of the data. YNK performed the treatment planning and conducted all planning evaluations. SHS, HSJ, JWJ, and SKY took care of the patients. SHS, HSJ, JWJ, SKY and CSK participated in the study design. SHS performed the statistical analysis and drafted the manuscript. All authors read and approved the final manuscript.
